# Influence of sex differences on microRNA gene regulation in disease

**DOI:** 10.1186/2042-6410-5-3

**Published:** 2014-02-01

**Authors:** Salil Sharma, Mansoureh Eghbali

**Affiliations:** 1 Department of Anesthesiology, Division of Molecular Medicine, and Cardiovascular Research Laboratories, David Geffen School of Medicine, University of California Los Angeles, BH-160CHS, Los Angeles, CA 90095-7115, USA

## Abstract

Sexual dimorphism is observed in most human diseases. The difference in the physiology and genetics between sexes can contribute tremendously to the disease prevalence, severity, and outcome. Both hormonal and genetic differences between males and females can lead to differences in gene expression patterns that can influence disease risk and course. MicroRNAs have emerged as potential regulatory molecules in all organisms. They can have a broad effect on every aspect of physiology, including embryogenesis, metabolism, and growth and development. Numerous microRNAs have been identified and elucidated to play a key role in cardiovascular diseases, as well as in neurological and autoimmune disorders. This is especially important as microRNA-based tools can be exploited as beneficial therapies for disease treatment and prevention. Sex steroid hormones as well as X-linked genes can have a considerable influence on the regulation of microRNAs. However, there are very few studies highlighting the role of microRNAs in sex biased diseases. This review attempts to summarize differentially regulated microRNAs in males versus females in different diseases and calls for more attention in this underexplored area that should set the basis for more effective therapeutic strategies for sexually dimorphic diseases.

## Introduction

Many complex regulatory steps are important in the conversion and dissemination of genetic information to molecular effectors. This regulation is fine tuned by a number of transcriptional and post transcriptional mechanisms. Sex differences can have major contributions in defining the course of these regulatory steps. Whether it is the presence of a Y chromosome, an extra X chromosome or a difference in the hormonal milieu, these gender differences are remarkably reflected in the disease outcomes. Gender differences have been already highlighted in a number of diseases and disorders associated with neurodevelopment, cancers, cardiovascular and immune systems [1-10]. MicroRNAs regulate various physiological processes ranging from cell growth and differentiation to cell metabolism, development, morphogenesis, maturation and apoptosis [11-21]. The dysregulation of microRNAs and consequently of their target genes has been widely linked to various pathologic conditions of the heart, lung and brain, as well as in many cancers [22-33]. Thus, it is worthwhile to examine their role in the context of sex differences and disease outcome.

There is a differential expression of microRNAs in males and females. This sex-biased expression of microRNAs has been observed both in invertebrates as well as in higher organisms [34-38]. The sex difference in the expression of microRNAs has also been observed in embryonic stem cell differentiation. In particular, it has been shown that miR-302 is enriched in the male embryonic stem cell differentiation and germ line determination, but not in female cells [39]. Another example is the sex-associated differential expression of microRNAs during lung development, which ultimately leads to differences in the structure and function of lungs between males and females [40]. This sex-biased difference in microRNA expression is of great biomedical interest. The contribution of differential microRNA expression in sexually dimorphic disease development has also been explored to some extent [41,42]. This review will highlight our current knowledge on the differential expression of microRNAs between genders, the sex chromosome and sex hormone-mediated regulation of microRNAs and how this sexually biased microRNA expression contributes to the sex differences in the disease outcome.

## Review

### MicroRNA biogenesis

MicroRNAs are small, non-coding, approximately 19-22-nucleotide-long RNA molecules. Their function is to regulate gene expression by mRNA degradation or to attenuate translation of their target genes. The very first identified microRNAs, lin-4 and let-7 were discovered in *Caenorhabditis elegans* in the early 1990s [43-45]. MicroRNAs are reported in a wide variety of organisms ranging from single-cell organisms to metazoans, plants and mammals [46-49]. In mammals, it is estimated that approximately 60% of all protein-coding genes are under microRNA regulation [50,51]. MicroRNAs are involved in the regulation of virtually all cellular processes including metabolism, development, morphogenesis, programmed cell death, angiogenesis and so on [14,21,33,52-57]. Their dysregulated expression is also associated with human pathologies, including neurological disorders, cardiovascular diseases and cancer [27,28,30].

With the help of RNA polymerase II, microRNAs are transcribed from long precursor molecules called pri-miRNA present as independent genes or in the introns of protein coding genes. Two RNA polymerase III enzymes, Drosha and Dicer, act on these hairpin pri-miRNAs. Drosha cleaves the hairpin into approximately 70 nucleotide pre-miRNA molecule, which then is exported out of the nucleus by active transport. In the cytosol, Dicer processes it to an approximately 20-bp miRNA/miRNA* duplex. One strand of this duplex represents the mature microRNA. This mature miRNA is then incorporated in a multi protein complex, the miRNA-induced silencing complex (miRISC). MicroRNA-induced translational repression is guided within miRISC. Lastly, the assembly and function of ribonucleases and miRISCs are assisted by proteins and key cofactors including argonaute proteins [43-45,58]. The regulation of microRNA biogenesis is important for the tissue- and development-specific control of microRNA expression. The sophisticated control of microRNA biogenesis, function and decay is essential, as these regulatory molecules play a fundamental role in all known cellular processes.

### Sex-specific difference in microRNA expression

Hormonal and genetic differences between males and females bear considerable impact on health and disease. One particular area of interest is the microRNAs which can have broad effect on downstream signaling pathways. Differences in miRNA expression between males and females can help us further understand the biological and physiological differences between the sexes. These sex differences in microRNA expression are a result of both hormonal and genetic differences between the sexes.

#### Sex hormonal regulation of microRNAs

Several sex steroid hormones, such as estradiol, progesterone and testosterone, regulate microRNA expression [58-64]. A distinct sex-specific pattern of microRNA expression in neonatal rodent brains was observed. This difference in expression almost disappeared when the conversion of testosterone to estradiol was blocked in males, suggesting that microRNAs are regulated by estrogens [65]. Sex hormones bind to nuclear hormone receptors to directly or indirectly alter gene expression. This ligand-bound nuclear hormone complex binds to the promoter elements of multiple genes, recruits coactivators or corepressors, and regulates gene transcription. MicroRNAs are either embedded in their host genes or have their own promoter elements. They are either coregulated or regulated in the same manner as their host genes. In addition, nuclear hormone receptors can regulate multiple downstream target genes. These target genes could induce or repress microRNAs as a part of a bigger molecular network [66-68]. Thus, the hormonal regulation of microRNAs could be direct as well as indirect. Another important mechanism of hormonal regulation of microRNAs is at the level of the microRNA processing machinery. There are multiple steps involved in the processing of microRNAs. The ribonucleases Drosha and Dicer are part of larger protein complexes, and play key roles in the activation or repression of these complexes to regulate microRNA maturation. Furthermore, hormones can also regulate Drosha and Dicer, thus broadly regulating microRNAs. For example, estradiol signaling has been shown to alter the expression of argonaute, Drosha and Dicer [69-73]. Thus, hormonal regulation of microRNAs can occur both directly by binding to the promoter elements of microRNAs or indirectly via regulation of post transcriptional processing proteins. This could have broad signaling effects at the molecular and cellular level.

#### Sex chromosomal regulation of microRNAs

Differences in the expression of microRNAs can be partly attributed to a differential regulation by sex chromosome genes. Gene expression in males and females differs since females possess two copies of the X chromosome and lack the Y chromosome. Since males are hemizygous for the X chromosome-linked genes, they are more prone to diseases associated with recessive mutations. There is a high density of microRNAs on the X chromosome. According to miRBase microRNA archive (http://www.miRBase.org 2013), the X chromosome encodes 113 microRNAs whereas Y chromosome encodes only 2. Many genes in the X chromosome are inactivated in order to balance the expression of most but not all X-linked genes in females and males. Approximately 15% of genes encoded by the inactive X chromosome in humans escape inactivation [74-77], which in some cases results in higher expression of the X gene in females than in males. The inactivation status of X-linked miRNAs is not well characterized. Interestingly, the expression of sex-biased microRNAs in the neonatal brain could stem from both hormone and sex chromosome effects [78].

### Sex-biased differences in microRNA expression influences disease pathogenesis

Males and females differ in disease outcome and pathogenesis. In a number of autoimmune diseases, females have higher incidence of disease than males [10,42,79,80]. In contrast, the pathogenesis of many cancers is higher in males than females [81]. The incidence of cardiovascular diseases is also higher in men than women before menopause [82,83]. Therefore, differential expression of microRNAs between the sexes may be an important underlying mechanism for gender-biased disease outcome (Table 1).

**Table 1:** MicroRNAs showing sex-biased expression in different pathological conditions

**Disease/development**	**MicroRNAs**	**Expression changes between males and females**	**Hormone regulated**	**Chromosome linked**
Autoimmune diseases				
Systemic lupus erythematosus	miR-182 cluster, miR-31, and miR-148a [84]	M < F	Estrogens	-
Neurodegenerative diseases				
Schizophrenia	miR-30b [85]	M > F	Estrogens	
	let-7f-2, miR-18b, miR-505, miR-502, miR-188, miR-325, miR-660 and miR-509-3 [41]	No expression changes reported (only mutational changes between disease and control)		X chromosome
Cerebral ischemia	miR-23a [37]	M < F	-	-
Neurodevelopment	miR-322, miR-574, and miR-873 [65]	M < F	Estrogens	-
Metabolic diseases	miR-221, let-7 g [86]	M < F	-	-
Breast cancer	miR17, let-7a [87];	M < F	-	-
	miR-137 [35]	M < F promoter methylation of miR-137	-	-
Liver fibrosis	miR-29a, miR-29b [88]	M < F	Estrogens	-

#### Autoimmune diseases

Autoimmunity results from the misdirected response of immune system to body’s healthy cells, leading to autoimmune diseases including rheumatoid arthritis, type I diabetes and systemic lupus erythematosus [89,90]. Multiple factors, hormonal or genetic, either protect males or increase vulnerability of females [10]. Behavioral differences between the sexes could also alter susceptibility to infection [91]. The X chromosome is enriched in microRNAs and there is a possibility that microRNAs may escape X-linked inactivation [78,92]. This could result in the suppression of many microRNA-regulated target genes involved in immunosuppressive pathways, leading to a heightened autoimmune response in females and their predisposition to autoimmune diseases [93,94]. Predisposition of women to systemic lupus erythematosus is due to overexpression of immune active genes, resulting in T cell autoreactivity. Various factors contribute to the pathogenesis of systemic lupus erythematosus. These include genetic factors such as skewed X chromosome inactivation, environmental and epigenetic factors such as DNA methylation and smoking, and hormones or microRNAs that could possibly make women more susceptible [95-97]. Interestingly, in the NZB/WF1 murine model that closely mimics human lupus, a clear sex difference in the systemic lupus erythematosus-associated microRNAs was observed. Particularly, increased expression of miR-182 cluster, miR-155, miR-31, and miR-148a was observed in female NZB/WF1 mice at an age after the onset of lupus. Administration of estrogens to castrated male NZB/WF1 mice resulted in the expression of female-associated systemic lupus erythematosus microRNAs including the miR-182 cluster, miR-379, and miR-148a, thus demonstrating the role of sex hormones in the regulation of sexual dimorphism of microRNAs [84]. In addition, estrogens are important modulators of the immune system and contribute to the gender difference in autoimmune disease susceptibility [98-100]. Estrogen treatment augments innate immune response of murine splenic lymphocytes to Lipopolysaccharide by manipulating select microRNAs, including miR146a and miR-223 [101]. Thus, estrogen-mediated regulation of microRNAs may serve as an important parameter in gender-biased disease susceptibility.

#### Neurodegenerative disorders

The precise temporal regulation of microRNAs is especially important in the development of the central nervous system, in which developmental events need to be finely tuned. This became evident when deletion of the microRNA processing enzyme Dicer in zebrafish resulted in the disruption of neuronal differentiation [21]. MicroRNAs are involved in various neurologic processes including neuronal development (miR-430, miR-9, miR-10) [21,102-106] and neuronal cell maintenance (miR-29a/b, miR-134) [107,108]. Also, of scientific interest, is the role of microRNAs in the aging brain since there is a global downregulation of gene expression in the brain with age [109-112]. It is also well known that the functional and cognitive decline in the aging brain differs between men and women, as many neurological disorders such as Alzheimer’s disease are expressed differentially between the sexes. Sex hormones and chromosomes could very well contribute to this difference [113-115]. MicroRNAs including miR-125a/b, miR-495 and miR-181 have been shown to be dysregulated in a variety of neurological disorders such as Alzheimer’s disease. These microRNAs also target proteins such as β-site amyloid precursor protein-cleaving enzyme-1 that are critical in the pathology of Alzheimer’s disease [116-118]. Hormonal regulation of microRNAs is attributed to neurodegenerative disorders [65]. It is also speculated that sex chromosomes could regulate microRNAs in neurodegenerative disorders [78].

The effects of ionizing radiation on the expression of microRNAs were studied in different brain regions in male and female mice. Interestingly, the number of microRNAs that showed changes in expression upon exposure to radiation was larger in females than males. Notably, the expression of miR-29a was downregulated only in the frontal cortex region of females accompanied by an increase in the protein levels of miR29a target DNMT3a, a DNA methyltransferase. It is possible that an increase in DNA methylation via miR29a downregulation serves as a protective mechanism in females against the adverse effects of radiation [34]. In contrast, only a few studies in humans have measured sex differences in microRNA expression in the brains of patients with neurological diseases such as Alzheimer’s disease or schizophrenia. The levels of miR-30b were observed to be significantly downregulated in the brains of female schizophrenic patients compared to males. MiR-30b was shown to be regulated by estrogens and therefore, it is most likely under hormonal control [85]. Another relevant study specifically focused on the association of microRNAs on the X chromosome with high prevalence of schizophrenia in males compared to females. Mutations in X-linked microRNAs that can possibly predispose males to schizophrenia were identified. Some key targets identified for these microRNAs include Neuregulin-1, Disrupted in schizophrenia-1, and Regulator of G-protein signaling-4, which have been previously described as candidate genes for schizophrenia [41]. The underlying cause of cerebral ischemia differs between males and females. In males the cause of stroke-related death is primarily due to caspase-independent mechanisms, whereas in females it involves caspase-dependent pathways [119-121]. X-linked inhibitor of apoptosis, which is the primary endogenous inhibitor of caspases, is significantly reduced in post-stroke females compared to no significant changes in males. This was attributed to the differential expression of the microRNA miR-23a in the male and female brains. Also, the X-linked inhibitor of apoptosis was characterized as the bona fide target of miR-23a, thus implicating miR23a as a candidate for sexually dimorphic diseases [37]. Many neurodevelopmental disorders including schizophrenia and autism are linked to prenatal stress [122,123]. In a murine model, early gestation was reported to be the period where male mouse embryos are most susceptible to maternal stress. As adults, these male mice showed cognitive defects typical of human neurodegenerative disease. They also showed dysmasculization in their response to stress, as well as a female pattern of gene expression associated with neurodevelopment. The microRNA profile in these male brains included significant downregulation of miR-322, miR-574, and miR-873, showing similarity to control female brains [65]. These data further confirm the important role of microRNAs in the development of the sexually dimorphic brain.

#### Metabolism

Metabolic syndrome includes all the risk factors that can increase the susceptibility to, or lead to health disorders, including heart disease, type 2 diabetes, and stroke. Because of high obesity rate, metabolic syndrome is fast approaching as one of the major risk factors for heart disease [124-126]. Sex is a significant modifier for metabolic syndrome-related cardiovascular and non-cardiovascular complications. Wang *et. al.* discovered that miR-221 and let- 7 g were expressed more prominently in the plasma of women than men manifesting metabolic syndrome [86]. This may have significant implications in the susceptibility of these women to cardiovascular risk factors. Interestingly, the expression of these microRNAs positively correlated with the increase in number of risk components associated with metabolic syndrome including high blood pressure and low HDL levels.

#### Cardiovascular diseases

The incidence and progression of heart disease is markedly higher in men than in age-matched women before menopause, whereas after menopause the incidence is similar or even higher in women than age-matched men [82,83]. The lower incidence and better protection of women against coronary artery disease during reproductive years is attributed to the presence of female sex hormones [127]. The protective role of estradiol has been highlighted in the context of myocardial ischemia/reperfusion injury in women as well as in females in animal models [128]. In addition, the regulation of microRNAs by estrogens and other sex hormones is well established [58-64]. However, the link between sex differences in microRNAs and their effect on the incidence and course of cardiovascular disease is largely unexplored. Future studies on this issue will help in the understanding of sex-biased difference in cardiovascular disease.

#### Cancer and liver disease

The dysregulation of microRNAs is implicated in the pathogenesis of many cancers. For instance, the regulation of microRNAs is involved in the progression of breast cancer. Breast cancer is prevalent among women in the western world, with much lower incidence among males [129-131]. Multiple microRNAs have dysregulated expression in breast cancer versus normal controls both in breast tissue and in blood circulation [132-135]. Of interest, Pinto *et. al.* presented a significant correlation between miR17 and let-7a frequency and regulation in familial breast cancer in women compared to men [87]. Langevin et. al. also show that promoter methylation of miR-137 occurs frequently in squamous cell carcinoma of the head and neck. This microRNA is implicated in cell cycle control and differentiation. This aberrant methylation shows a bias toward females and may be associated with environmental and personal risk factors [35].

Some chronic liver diseases manifest different progression rates in males versus females. For example, liver fibrosis is one of the hallmarks of chronic viral hepatitis and has a higher incidence in males than females. It is characterized by extensive deposition of collagens in the extracellular matrix [136,137]. In the carbon tetrachloride mouse model of fibrosis, females are more protected against liver fibrosis than males. This has been attributed to the induction of miR-29a and miR-29b by estrogens in females. MiR-29 family members decrease the content of collagens by directly targeting them and thus block the process of development of fibrosis [88,138].

## Conclusions

In recent years, microRNAs have emerged as powerful regulatory molecules. Gain and loss of function studies in various animal models mimicking human disease have shed light on the functional role and target specification of microRNAs. However, the role of miRNAs in mediating sex biases in diseases is understudied and under-appreciated. There is a plethora of literature showing sexual dimorphism in the context of neurological and autoimmunity disorders. This is attributed to both hormonal and sex chromosome differences between males and females. Estrogens have been shown to regulate a number of microRNAs in various cell studies. Similarly, the X chromosome is enriched in a number of microRNAs involved in various physiological processes, especially immunity. Thus, it is likely that these microRNAs can influence sex-specific responses to disease prevalence, pathogenesis, and outcome. Further research in exploring the gender-specific roles of microRNAs is extremely important for the development of effective therapeutic strategies for sexually dimorphic diseases.

## Competing interests

The authors declare that they have no competing interests.

## Authors’ contributions

SS and ME drafted the manuscript. Both authors read and approved the final manuscript.

## Authors’ information

http://www.anes.ucla.edu/dmm/eghbali/.

## References

[B1] Czlonkowska A Ciesielska A Gromadzka G Kurkowska-Jastrzebska I Gender differences in neurological disease: role of estrogens and cytokines Endocrine 2006 5 243 256 10.1385/ENDO:29:2:243 16785600

[B2] McCarthy MM Arnold AP Ball GF Blaustein JD De Vries GJ Sex differences in the brain: the not so inconvenient truth J Neurosci 2012 5 2241 2247 10.1523/JNEUROSCI.5372-11.2012 22396398 PMC3295598

[B3] Dorak MT Karpuzoglu E Gender differences in cancer susceptibility: an inadequately addressed issue Front Genet 2012 5 268 23226157 10.3389/fgene.2012.00268 PMC3508426

[B4] Majek O Gondos A Jansen L Emrich K Holleczek B Katalinic A Nennecke A Eberle A Brenner H Sex differences in colorectal cancer survival: population-based analysis of 164,996 colorectal cancer patients in Germany PLoS One 2013 5 e68077 10.1371/journal.pone.0068077 23861851 PMC3702575

[B5] Maas AH Appelman YE Gender differences in coronary heart disease Neth Heart J 2010 5 598 602 10.1007/s12471-010-0841-y PMC3018605 21301622

[B6] Wenger NK Gender disparity in cardiovascular disease: bias or biology? Expert Rev Cardiovasc Ther 2012 5 1401 1411 10.1586/erc.12.133 23244361

[B7] Palaszynski KM Liu H Loo KK Voskuhl RR Estriol treatment ameliorates disease in males with experimental autoimmune encephalomyelitis: implications for multiple sclerosis J Neuroimmunol 2004 5 84 89 10.1016/j.jneuroim.2003.12.015 15020068

[B8] Palaszynski KM Loo KK Ashouri JF Liu HB Voskuhl RR Androgens are protective in experimental autoimmune encephalomyelitis: implications for multiple sclerosis J Neuroimmunol 2004 5 144 152 10.1016/j.jneuroim.2003.11.004 14698857

[B9] Smith-Bouvier DL Divekar AA Sasidhar M Du S Tiwari-Woodruff SK King JK Arnold AP Singh RR Voskuhl RR A role for sex chromosome complement in the female bias in autoimmune disease J Exp Med 2008 5 1099 1108 10.1084/jem.20070850 18443225 PMC2373842

[B10] Voskuhl R Sex differences in autoimmune diseases Biol Sex Differ 2011 5 1 10.1186/2042-6410-2-1 21208397 PMC3022636

[B11] Chen JF Mandel EM Thomson JM Wu Q Callis TE Hammond SM Conlon FL Wang DZ The role of microRNA-1 and microRNA-133 in skeletal muscle proliferation and differentiation Nat Genet 2006 5 228 233 10.1038/ng1725 16380711 PMC2538576

[B12] Carraro G El-Hashash A Guidolin D Tiozzo C Turcatel G Young BM De Langhe SP Bellusci S Shi W Parnigotto PP Warburton D miR-17 family of microRNAs controls FGF10-mediated embryonic lung epithelial branching morphogenesis through MAPK14 and STAT3 regulation of E-Cadherin distribution Dev Biol 2009 5 238 250 10.1016/j.ydbio.2009.06.020 19559694 PMC2735610

[B13] Chen CZ Li L Lodish HF Bartel DP MicroRNAs modulate hematopoietic lineage differentiation Science 2004 5 83 86 10.1126/science.1091903 14657504

[B14] Esau C Davis S Murray SF Yu XX Pandey SK Pear M Watts L Booten SL Graham M McKay R Subramaniam A Propp S Lollo BA Freier S Bennett CF Bhanot S Monia BP miR-122 regulation of lipid metabolism revealed by in vivo antisense targeting Cell Metab 2006 5 87 98 10.1016/j.cmet.2006.01.005 16459310

[B15] Jovanovic M Hengartner MO miRNAs and apoptosis: RNAs to die for Oncogene 2006 5 6176 6187 10.1038/sj.onc.1209912 17028597

[B16] Karrich JJ Jachimowski LC Libouban M Iyer A Brandwijk K Taanman-Kueter EW Nagasawa M de Jong EC Uittenbogaart CH Blom B MicroRNA-146a regulates survival and maturation of human plasmacytoid dendritic cells Blood 2013 5 3001 3009 10.1182/blood-2012-12-475087 24014244 PMC3811175

[B17] Naguibneva I meyar-Zazoua M Polesskaya A it-Si-Ali S Groisman R Souidi M Cuvellier S Harel-Bellan A The microRNA miR-181 targets the homeobox protein Hox-A11 during mammalian myoblast differentiation Nat Cell Biol 2006 5 278 284 10.1038/ncb1373 16489342

[B18] Poy MN Eliasson L Krutzfeldt J Kuwajima S Ma X Macdonald PE Pfeffer S Tuschl T Rajewsky N Rorsman P Stoffel M A pancreatic islet-specific microRNA regulates insulin secretion Nature 2004 5 226 230 10.1038/nature03076 15538371

[B19] Watanabe M Kinutani M Naito M Ochi O Takashima Y Distribution analysis of transferred donor cells in avian blastodermal chimeras Development 1992 5 331 338 1591995 10.1242/dev.114.2.331

[B20] Zhou Y Jiang H Gu J Tang Y Shen N Jin Y MicroRNA-195 targets ADP-ribosylation factor-like protein 2 to induce apoptosis in human embryonic stem cell-derived neural progenitor cells Cell Death Dis 2013 5 e695 10.1038/cddis.2013.195 23807224 PMC3702293

[B21] Giraldez AJ Cinalli RM Glasner ME Enright AJ Thomson JM Baskerville S Hammond SM Bartel DP Schier AF MicroRNAs regulate brain morphogenesis in zebrafish Science 2005 5 833 838 10.1126/science.1109020 15774722

[B22] Abe M Bonini NM MicroRNAs and neurodegeneration: role and impact Trends Cell Biol 2013 5 30 36 10.1016/j.tcb.2012.08.013 23026030 PMC3540990

[B23] Croce CM Causes and consequences of microRNA dysregulation in cancer Nat Rev Genet 2009 5 704 714 10.1038/nrg2634 19763153 PMC3467096

[B24] Dvinge H Git A Graf S Salmon-Divon M Curtis C Sottoriva A Zhao Y Hirst M Armisen J Miska EA Chin SF Provenzano E Turashvili G Green A Ellis I Aparicio S Caldas C The shaping and functional consequences of the microRNA landscape in breast cancer Nature 2013 5 378 382 10.1038/nature12108 23644459

[B25] Kuss AW Chen W MicroRNAs in brain function and disease Curr Neurol Neurosci Rep 2008 5 190 197 10.1007/s11910-008-0031-0 18541114

[B26] Pagdin T Lavender P MicroRNAs in lung diseases Thorax 2012 5 183 184 10.1136/thoraxjnl-2011-200532 21836155

[B27] Persengiev SP Kondova II Bontrop RE The Impact of MicroRNAs on Brain Aging and Neurodegeneration Curr Gerontol Geriatr Res 2012 5 359369 22312330 10.1155/2012/359369 PMC3270527

[B28] Sassen S Miska EA Caldas C MicroRNA: implications for cancer Virchows Arch 2008 5 1 10 18040713 10.1007/s00428-007-0532-2 PMC2151131

[B29] Sessa R Hata A Role of microRNAs in lung development and pulmonary diseases Pulm Circ 2013 5 315 328 10.4103/2045-8932.114758 24015331 PMC3757825

[B30] Small EM Frost RJ Olson EN MicroRNAs add a new dimension to cardiovascular disease Circulation 2010 5 1022 1032 10.1161/CIRCULATIONAHA.109.889048 20194875 PMC2847432

[B31] Small EM Olson EN Pervasive roles of microRNAs in cardiovascular biology Nature 2011 5 336 342 10.1038/nature09783 21248840 PMC3073349

[B32] Sonntag KC Woo TU Krichevsky AM Converging miRNA functions in diverse brain disorders: a case for miR-124 and miR-126 Exp Neurol 2012 5 427 435 10.1016/j.expneurol.2011.11.035 22178324 PMC3335933

[B33] Stahlhut Espinosa CE Slack FJ The role of microRNAs in cancer Yale J Biol Med 2006 5 131 140 17940623 PMC1994807

[B34] Koturbash I Zemp F Kolb B Kovalchuk O Sex-specific radiation-induced microRNAome responses in the hippocampus, cerebellum and frontal cortex in a mouse model Mutat Res 2011 5 114 118 10.1016/j.mrgentox.2010.05.007 20478395

[B35] Langevin SM Stone RA Bunker CH Grandis JR Sobol RW Taioli E MicroRNA-137 promoter methylation in oral rinses from patients with squamous cell carcinoma of the head and neck is associated with gender and body mass index Carcinogenesis 2010 5 864 870 10.1093/carcin/bgq051 20197299 PMC2864416

[B36] Marco A Kozomara A Hui JH Emery AM Rollinson D Griffiths-Jones S Ronshaugen M Sex-biased expression of microRNAs in *Schistosoma mansoni* PLoS Negl Trop Dis 2013 5 e2402 10.1371/journal.pntd.0002402 24069470 PMC3772069

[B37] Siegel C Li J Liu F Benashski SE McCullough LD miR-23a regulation of X-linked inhibitor of apoptosis (XIAP) contributes to sex differences in the response to cerebral ischemia Proc Natl Acad Sci USA 2011 5 11662 11667 10.1073/pnas.1102635108 21709246 PMC3136267

[B38] Wu W Ren Q Li C Wang Y Sang M Zhang Y Li B Characterization and comparative profiling of MicroRNAs in a sexual dimorphism insect, *Eupolyphaga sinensis* Walker PLoS One 2013 5 e59016 10.1371/journal.pone.0059016 23620723 PMC3631196

[B39] Ciaudo C Servant N Cognat V Sarazin A Kieffer E Viville S Colot V Barillot E Heard E Voinnet O Highly dynamic and sex-specific expression of microRNAs during early ES cell differentiation PLoS Genet 2009 5 e1000620 10.1371/journal.pgen.1000620 19714213 PMC2725319

[B40] Mujahid S Logvinenko T Volpe MV Nielsen HC miRNA regulated pathways in late stage murine lung development BMC Dev Biol 2013 5 13 10.1186/1471-213X-13-13 23617334 PMC3644234

[B41] Feng J Sun G Yan J Noltner K Li W Buzin CH Longmate J Heston LL Rossi J Sommer SS Evidence for X-chromosomal schizophrenia associated with microRNA alterations PLoS One 2009 5 e6121 10.1371/journal.pone.0006121 19568434 PMC2699475

[B42] Hewagama A Gorelik G Patel D Liyanarachchi P McCune WJ Somers E Gonzalez-Rivera T Strickland F Richardson B Overexpression of X-linked genes in T cells from women with lupus J Autoimmun 2013 5 60 71 23434382 10.1016/j.jaut.2012.12.006 PMC3622754

[B43] Bartel DP MicroRNAs: target recognition and regulatory functions Cell 2009 5 215 233 10.1016/j.cell.2009.01.002 19167326 PMC3794896

[B44] Chekulaeva M Filipowicz W Mechanisms of miRNA-mediated post-transcriptional regulation in animal cells Curr Opin Cell Biol 2009 5 452 460 10.1016/j.ceb.2009.04.009 19450959

[B45] Carthew RW Sontheimer EJ Origins and Mechanisms of miRNAs and siRNAs Cell 2009 5 642 655 10.1016/j.cell.2009.01.035 19239886 PMC2675692

[B46] Molnar A Schwach F Studholme DJ Thuenemann EC Baulcombe DC miRNAs control gene expression in the single-cell alga *Chlamydomonas reinhardtii* Nature 2007 5 1126 1129 10.1038/nature05903 17538623

[B47] Tarver JE Sperling EA Nailor A Heimberg AM Robinson JM King BL Pisani D Donoghue PC Peterson KJ miRNAs: Small Genes with Big Potential in Metazoan Phylogenetics Mol Biol Evol 2013 5 2369 2382 10.1093/molbev/mst133 23913097

[B48] Voinnet O Origin, biogenesis, and activity of plant microRNAs Cell 2009 5 669 687 10.1016/j.cell.2009.01.046 19239888

[B49] Kim VN Han J Siomi MC Biogenesis of small RNAs in animals Nat Rev Mol Cell Biol 2009 5 126 139 10.1038/nrm2632 19165215

[B50] Fabian MR Sonenberg N Filipowicz W Regulation of mRNA translation and stability by microRNAs Annu Rev Biochem 2010 5 351 379 10.1146/annurev-biochem-060308-103103 20533884

[B51] Friedman RC Farh KK Burge CB Bartel DP Most mammalian mRNAs are conserved targets of microRNAs Genome Res 2009 5 92 105 18955434 10.1101/gr.082701.108 PMC2612969

[B52] Mondol V Pasquinelli AE Let’s make it happen: the role of let-7 microRNA in development Curr Top Dev Biol 2012 5 1 30 22365733 10.1016/B978-0-12-387038-4.00001-X

[B53] Hornstein E Shomron N Canalization of development by microRNAs Nat Genet 2006 5 S20 S24 16736020 10.1038/ng1803

[B54] DiCarlo LA Jenkins JM Kriegler C Intraventricular electrogram analysis for discrimination of ventricular tachycardia from ventricular fibrillation J Electrocardiol 1992 5 135 1552245 10.1016/s0022-0736(10)80035-4

[B55] Baehrecke EH miRNAs: micro managers of programmed cell death Curr Biol 2003 5 R473 R475 10.1016/S0960-9822(03)00405-6 12814564

[B56] Suarez Y Sessa WC MicroRNAs as novel regulators of angiogenesis Circ Res 2009 5 442 454 10.1161/CIRCRESAHA.108.191270 19246688 PMC2760389

[B57] Landskroner-Eiger S Moneke I Sessa WC miRNAs as modulators of angiogenesis Cold Spring Harb Perspect Med 2013 5 a006643 23169571 10.1101/cshperspect.a006643 PMC3552340

[B58] Lam EW Shah K Brosens JJ The diversity of sex steroid action: the role of micro-RNAs and FOXO transcription factors in cycling endometrium and cancer J Endocrinol 2012 5 13 25 10.1530/JOE-10-0480 21382987

[B59] Bhat-Nakshatri P Wang G Collins NR Thomson MJ Geistlinger TR Carroll JS Brown M Hammond S Srour EF Liu Y Nakshatri H Estradiol-regulated microRNAs control estradiol response in breast cancer cells Nucleic Acids Res 2009 5 4850 4861 10.1093/nar/gkp500 19528081 PMC2724297

[B60] Klinge CM miRNAs and estrogen action Trends Endocrinol Metab 2012 5 223 233 10.1016/j.tem.2012.03.002 22503553 PMC3348384

[B61] Kuokkanen S Chen B Ojalvo L Benard L Santoro N Pollard JW Genomic profiling of microRNAs and messenger RNAs reveals hormonal regulation in microRNA expression in human endometrium Biol Reprod 2010 5 791 801 10.1095/biolreprod.109.081059 19864316 PMC2842492

[B62] Paris O Ferraro L Grober OM Ravo M De Filippo MR Giurato G Nassa G Tarallo R Cantarella C Rizzo F Di BA Mottolese M Benes V Ambrosino C Nola E Weisz A Direct regulation of microRNA biogenesis and expression by estrogen receptor beta in hormone-responsive breast cancer Oncogene 2012 5 4196 4206 10.1038/onc.2011.583 22231442

[B63] Waltering KK Porkka KP Jalava SE Urbanucci A Kohonen PJ Latonen LM Kallioniemi OP Jenster G Visakorpi T Androgen regulation of micro-RNAs in prostate cancer Prostate 2011 5 604 614 10.1002/pros.21276 20945501

[B64] Wang WL Chatterjee N Chittur SV Welsh J Tenniswood MP Effects of 1alpha,25 dihydroxyvitamin D3 and testosterone on miRNA and mRNA expression in LNCaP cells Mol Cancer 2011 5 58 10.1186/1476-4598-10-58 21592394 PMC3112430

[B65] Morgan CP Bale TL Early prenatal stress epigenetically programs dysmasculinization in second-generation offspring via the paternal lineage J Neurosci 2011 5 11748 11755 10.1523/JNEUROSCI.1887-11.2011 21849535 PMC3164823

[B66] Kininis M Kraus WL A global view of transcriptional regulation by nuclear receptors: gene expression, factor localization, and DNA sequence analysis Nucl Recept Signal 2008 5 e005 18301785 10.1621/nrs.06005 PMC2254333

[B67] Kato S Yokoyama A Fujiki R Nuclear receptor coregulators merge transcriptional coregulation with epigenetic regulation Trends Biochem Sci 2011 5 272 281 10.1016/j.tibs.2011.01.001 21315607

[B68] Castellano L Giamas G Jacob J Coombes RC Lucchesi W Thiruchelvam P Barton G Jiao LR Wait R Waxman J Hannon GJ Stebbing J The estrogen receptor-alpha-induced microRNA signature regulates itself and its transcriptional response Proc Natl Acad Sci USA 2009 5 15732 15737 10.1073/pnas.0906947106 19706389 PMC2747188

[B69] Gregory RI Yan KP Amuthan G Chendrimada T Doratotaj B Cooch N Shiekhattar R The Microprocessor complex mediates the genesis of microRNAs Nature 2004 5 235 240 10.1038/nature03120 15531877

[B70] Siomi H Siomi MC Posttranscriptional regulation of microRNA biogenesis in animals Mol Cell 2010 5 323 332 10.1016/j.molcel.2010.03.013 20471939

[B71] Michlewski G Guil S Semple CA Caceres JF Posttranscriptional regulation of miRNAs harboring conserved terminal loops Mol Cell 2008 5 383 393 10.1016/j.molcel.2008.10.013 18995836 PMC2631628

[B72] Yamagata K Fujiyama S Ito S Ueda T Murata T Naitou M Takeyama K Minami Y O’Malley BW Kato S Maturation of microRNA is hormonally regulated by a nuclear receptor Mol Cell 2009 5 340 347 10.1016/j.molcel.2009.08.017 19854141

[B73] Macias S Michlewski G Caceres JF Hormonal regulation of microRNA biogenesis Mol Cell 2009 5 172 173 10.1016/j.molcel.2009.10.006 19854126

[B74] Khil PP Smirnova NA Romanienko PJ Camerini-Otero RD The mouse X chromosome is enriched for sex-biased genes not subject to selection by meiotic sex chromosome inactivation Nat Genet 2004 5 642 646 10.1038/ng1368 15156144

[B75] Straub T Becker PB Dosage compensation: the beginning and end of generalization Nat Rev Genet 2007 5 47 57 17173057 10.1038/nrg2013

[B76] Carrel L Willard HF X-inactivation profile reveals extensive variability in X-linked gene expression in females Nature 2005 5 400 404 10.1038/nature03479 15772666

[B77] Song R Ro S Michaels JD Park C McCarrey JR Yan W Many X-linked microRNAs escape meiotic sex chromosome inactivation Nat Genet 2009 5 488 493 10.1038/ng.338 19305411 PMC2723799

[B78] Morgan CP Bale TL Sex differences in microRNA regulation of gene expression: no smoke, just miRs Biol Sex Differ 2012 5 22 10.1186/2042-6410-3-22 23009289 PMC3507674

[B79] Koch-Henriksen N Sorensen PS The changing demographic pattern of multiple sclerosis epidemiology Lancet Neurol 2010 5 520 532 10.1016/S1474-4422(10)70064-8 20398859

[B80] Amur S Parekh A Mummaneni P Sex differences and genomics in autoimmune diseases J Autoimmun 2012 5 J254 J265 10.1016/j.jaut.2011.12.001 22204900

[B81] Cook MB McGlynn KA Devesa SS Freedman ND Anderson WF Sex disparities in cancer mortality and survival Cancer Epidemiol Biomarkers Prev 2011 5 1629 1637 10.1158/1055-9965.EPI-11-0246 21750167 PMC3153584

[B82] Barrett-Connor E Sex differences in coronary heart disease. Why are women so superior? The 1995 Ancel Keys Lecture Circulation 1997 5 252 264 10.1161/01.CIR.95.1.252 8994444

[B83] Crabbe DL Dipla K Ambati S Zafeiridis A Gaughan JP Houser SR Margulies KB Gender differences in post-infarction hypertrophy in end-stage failing hearts J Am Coll Cardiol 2003 5 300 306 12535826 10.1016/s0735-1097(02)02710-9

[B84] Dai R McReynolds S Leroith T Heid B Liang Z Ahmed SA Sex differences in the expression of lupus-associated miRNAs in splenocytes from lupus-prone NZB/WF1 mice Biol Sex Differ 2013 5 19 10.1186/2042-6410-4-19 24175965 PMC3843556

[B85] Mellios N Galdzicka M Ginns E Baker SP Rogaev E Xu J Akbarian S Gender-specific reduction of estrogen-sensitive small RNA, miR-30b, in subjects with schizophrenia Schizophr Bull 2012 5 433 443 10.1093/schbul/sbq091 20732949 PMC3329977

[B86] Wang YT Tsai PC Liao YC Hsu CY Juo SH Circulating microRNAs have a sex-specific association with metabolic syndrome J Biomed Sci 2013 5 72 10.1186/1423-0127-20-72 24093444 PMC3851553

[B87] Pinto R Pilato B Ottini L Lambo R Simone G Paradiso A Tommasi S Different methylation and microRNA expression pattern in male and female familial breast cancer J Cell Physiol 2013 5 1264 1269 10.1002/jcp.24281 23160909

[B88] Zhang Y Wu L Wang Y Zhang M Li L Zhu D Li X Gu H Zhang CY Zen K Protective role of estrogen-induced miRNA-29 expression in carbon tetrachloride-induced mouse liver injury J Biol Chem 2012 5 14851 14862 10.1074/jbc.M111.314922 22393047 PMC3340269

[B89] Davidson A Diamond B Autoimmune diseases N Engl J Med 2001 5 340 350 10.1056/NEJM200108023450506 11484692

[B90] Cho JH Gregersen PK Genomics and the multifactorial nature of human autoimmune disease N Engl J Med 2011 5 1612 1623 10.1056/NEJMra1100030 22029983

[B91] Klein SL The effects of hormones on sex differences in infection: from genes to behavior Neurosci Biobehav Rev 2000 5 627 638 10.1016/S0149-7634(00)00027-0 10940438

[B92] Hewagama A Role of X-chromosome encoded miRNAs in autoimmunity: suppressing the suppressor and female predisposition Rheumatology: Current Research 2013 5 1000118

[B93] Klein SL Implications of X-linked gene regulation for sex differences in disease pathogenesis Bioessays 2011 5 789 790 10.1002/bies.201100128 21953595

[B94] Pinheiro I Dejager L Libert C X-chromosome-located microRNAs in immunity: might they explain male/female differences? The X chromosome-genomic context may affect X-located miRNAs and downstream signaling, thereby contributing to the enhanced immune response of females Bioessays 2011 5 791 802 10.1002/bies.201100047 21953569

[B95] Lockshin MD Biology of the sex and age distribution of systemic lupus erythematosus Arthritis Rheum 2007 5 608 611 10.1002/art.22676 17471529

[B96] Crispin JC Liossis SN Kis-Toth K Lieberman LA Kyttaris VC Juang YT Tsokos GC Pathogenesis of human systemic lupus erythematosus: recent advances Trends Mol Med 2010 5 47 57 10.1016/j.molmed.2009.12.005 20138006 PMC2823952

[B97] Tiffin N Adeyemo A Okpechi I A diverse array of genetic factors contribute to the pathogenesis of systemic lupus erythematosus Orphanet J Rare Dis 2013 5 2 10.1186/1750-1172-8-2 23289717 PMC3551738

[B98] Cutolo M Capellino S Sulli A Serioli B Secchi ME Villaggio B Straub RH Estrogens and autoimmune diseases Ann NY Acad Sci 2006 5 538 547 10.1196/annals.1386.043 17261796

[B99] Jansson L Holmdahl R Estrogen-mediated immunosuppression in autoimmune diseases Inflamm Res 1998 5 290 301 10.1007/s000110050332 9719493

[B100] Cuchacovich M Gatica H Tchernitchin AN [Role of sex hormones in autoimmune diseases] Rev Med Chil 1993 5 1045 1052 8191156

[B101] Dai R Phillips RA Zhang Y Khan D Crasta O Ahmed SA Suppression of LPS-induced interferon-gamma and nitric oxide in splenic lymphocytes by select estrogen-regulated microRNAs: a novel mechanism of immune modulation Blood 2008 5 4591 4597 10.1182/blood-2008-04-152488 18791161 PMC2597130

[B102] Plasterk RH Micro RNAs in animal development Cell 2006 5 877 881 10.1016/j.cell.2006.02.030 16530032

[B103] Delaloy C Liu L Lee JA Su H Shen F Yang GY Young WL Ivey KN Gao FB MicroRNA-9 coordinates proliferation and migration of human embryonic stem cell-derived neural progenitors Cell Stem Cell 2010 5 323 335 10.1016/j.stem.2010.02.015 20362537 PMC2851637

[B104] Krichevsky AM King KS Donahue CP Khrapko K Kosik KS A microRNA array reveals extensive regulation of microRNAs during brain development RNA 2003 5 1274 1281 10.1261/rna.5980303 13130141 PMC1370491

[B105] Li Y Wang F Lee JA Gao FB MicroRNA-9a ensures the precise specification of sensory organ precursors in Drosophila Genes Dev 2006 5 2793 2805 10.1101/gad.1466306 17015424 PMC1619947

[B106] Woltering JM Durston AJ MiR-10 represses HoxB1a and HoxB3a in zebrafish PLoS One 2008 5 e1396 10.1371/journal.pone.0001396 18167555 PMC2148072

[B107] Lippi G Steinert JR Marczylo EL D’Oro S Fiore R Forsythe ID Schratt G Zoli M Nicotera P Young KW Targeting of the Arpc3 actin nucleation factor by miR-29a/b regulates dendritic spine morphology J Cell Biol 2011 5 889 904 10.1083/jcb.201103006 21930776 PMC3207289

[B108] Schratt GM Tuebing F Nigh EA Kane CG Sabatini ME Kiebler M Greenberg ME A brain-specific microRNA regulates dendritic spine development Nature 2006 5 283 289 10.1038/nature04367 16421561

[B109] Li N Bates DJ An J Terry DA Wang E Up-regulation of key microRNAs, and inverse down-regulation of their predicted oxidative phosphorylation target genes, during aging in mouse brain Neurobiol Aging 2011 5 944 955 10.1016/j.neurobiolaging.2009.04.020 19487051

[B110] Lee CK Weindruch R Prolla TA Gene-expression profile of the ageing brain in mice Nat Genet 2000 5 294 297 10.1038/77046 10888876

[B111] Kumar A Gibbs JR Beilina A Dillman A Kumaran R Trabzuni D Ryten M Walker R Smith C Traynor BJ Hardy J Singleton AB Cookson MR Age-associated changes in gene expression in human brain and isolated neurons Neurobiol Aging 2013 5 1199 1209 10.1016/j.neurobiolaging.2012.10.021 23177596 PMC3545059

[B112] Inukai S de LA Turner M Slack F Novel microRNAs differentially expressed during aging in the mouse brain PLoS One 2012 5 e40028 10.1371/journal.pone.0040028 22844398 PMC3402511

[B113] McPherson S Back C Buckwalter JG Cummings JL Gender-related cognitive deficits in Alzheimer’s disease Int Psychogeriatr 1999 5 117 122 10.1017/S1041610299005670 11475426

[B114] Weissman MM Bland RC Canino GJ Faravelli C Greenwald S Hwu HG Joyce PR Karam EG Lee CK Lellouch J Lepine JP Newman SC Rubio-Stipec M Wells JE Wickramaratne PJ Wittchen H Yeh EK Cross-national epidemiology of major depression and bipolar disorder JAMA 1996 5 293 299 10.1001/jama.1996.03540040037030 8656541

[B115] Brookmeyer R Gray S Kawas C Projections of Alzheimer’s disease in the United States and the public health impact of delaying disease onset Am J Public Health 1998 5 1337 1342 10.2105/AJPH.88.9.1337 9736873 PMC1509089

[B116] Hebert SS Horre K Nicolai L Papadopoulou AS Mandemakers W Silahtaroglu AN Kauppinen S Delacourte A De SB Loss of microRNA cluster miR-29a/b-1 in sporadic Alzheimer’s disease correlates with increased BACE1/beta-secretase expression Proc Natl Acad Sci USA 2008 5 6415 6420 10.1073/pnas.0710263105 18434550 PMC2359789

[B117] Holsinger RM McLean CA Beyreuther K Masters CL Evin G Increased expression of the amyloid precursor beta-secretase in Alzheimer’s disease Ann Neurol 2002 5 783 786 10.1002/ana.10208 12112088

[B118] Somel M Guo S Fu N Yan Z Hu HY Xu Y Yuan Y Ning Z Hu Y Menzel C Hu H Lachmann M Zeng R Chen W Khaitovich P MicroRNA, mRNA, and protein expression link development and aging in human and macaque brain Genome Res 2010 5 1207 1218 10.1101/gr.106849.110 20647238 PMC2928499

[B119] Li J McCullough LD Sex differences in minocycline-induced neuroprotection after experimental stroke J Cereb Blood Flow Metab 2009 5 670 674 10.1038/jcbfm.2009.3 19190654 PMC2855881

[B120] Liu F Li Z Li J Siegel C Yuan R McCullough LD Sex differences in caspase activation after stroke Stroke 2009 5 1842 1848 10.1161/STROKEAHA.108.538686 19265047 PMC2674515

[B121] McCullough LD Zeng Z Blizzard KK Debchoudhury I Hurn PD Ischemic nitric oxide and poly (ADP-ribose) polymerase-1 in cerebral ischemia: male toxicity, female protection J Cereb Blood Flow Metab 2005 5 502 512 10.1038/sj.jcbfm.9600059 15689952

[B122] Meyer U Feldon J Dammann O Schizophrenia and autism: both shared and disorder-specific pathogenesis via perinatal inflammation? Pediatr Res 2011 5 26R 33R 10.1203/PDR.0b013e318212c196 21289540 PMC3086802

[B123] Kinney DK Munir KM Crowley DJ Miller AM Prenatal stress and risk for autism Neurosci Biobehav Rev 2008 5 1519 1532 10.1016/j.neubiorev.2008.06.004 18598714 PMC2632594

[B124] Huang PL A comprehensive definition for metabolic syndrome Dis Model Mech 2009 5 231 237 10.1242/dmm.001180 19407331 PMC2675814

[B125] Mottillo S Filion KB Genest J Joseph L Pilote L Poirier P Rinfret S Schiffrin EL Eisenberg MJ The metabolic syndrome and cardiovascular risk a systematic review and meta-analysis J Am Coll Cardiol 2010 5 1113 1132 10.1016/j.jacc.2010.05.034 20863953

[B126] Zambon A Pauletto P Crepaldi G Review article: the metabolic syndrome--a chronic cardiovascular inflammatory condition Aliment Pharmacol Ther 2005 5 20 23 16225466 10.1111/j.1365-2036.2005.02589.x

[B127] Hayward CS Kelly RP Collins P The roles of gender, the menopause and hormone replacement on cardiovascular function Cardiovasc Res 2000 5 28 49 10.1016/S0008-6363(00)00005-5 10727651

[B128] Murphy E Steenbergen C Gender-based differences in mechanisms of protection in myocardial ischemia-reperfusion injury Cardiovasc Res 2007 5 478 486 10.1016/j.cardiores.2007.03.025 17466956

[B129] Giordano SH Cohen DS Buzdar AU Perkins G Hortobagyi GN Breast carcinoma in men: a population-based study Cancer 2004 5 51 57 10.1002/cncr.20312 15221988

[B130] Hutchinson L Breast cancer: challenges, controversies, breakthroughs Nat Rev Clin Oncol 2010 5 669 670 10.1038/nrclinonc.2010.192 21116236

[B131] Weigelt B Peterse JL van V Breast cancer metastasis: markers and models Nat Rev Cancer 2005 5 591 602 10.1038/nrc1670 16056258

[B132] Blenkiron C Goldstein LD Thorne NP Spiteri I Chin SF Dunning MJ Barbosa-Morais NL Teschendorff AE Green AR Ellis IO Tavare S Caldas C Miska EA MicroRNA expression profiling of human breast cancer identifies new markers of tumor subtype Genome Biol 2007 5 R214 10.1186/gb-2007-8-10-r214 17922911 PMC2246288

[B133] Chan M Liaw CS Ji SM Tan HH Wong CY Thike AA Tan PH Ho GH Lee AS Identification of circulating microRNA signatures for breast cancer detection Clin Cancer Res 2013 5 4477 4487 10.1158/1078-0432.CCR-12-3401 23797906

[B134] Iorio MV Ferracin M Liu CG Veronese A Spizzo R Sabbioni S Magri E Pedriali M Fabbri M Campiglio M Menard S Palazzo JP Rosenberg A Musiani P Volinia S Nenci I Calin GA Querzoli P Negrini M Croce CM MicroRNA gene expression deregulation in human breast cancer Cancer Res 2005 5 7065 7070 10.1158/0008-5472.CAN-05-1783 16103053

[B135] Mulrane L McGee SF Gallagher WM O’Connor DP miRNA dysregulation in breast cancer Cancer Res 2013 5 6554 6562 10.1158/0008-5472.CAN-13-1841 24204025

[B136] Khairy M bdel-Rahman M El-Raziky M El-Akel W Zayed N Khatab H Esmat G Non-invasive prediction of hepatic fibrosis in patients with chronic HCV based on the routine pre-treatment workup Hepat Mon 2012 5 e6718 23346149 10.5812/hepatmon.6718 PMC3549416

[B137] Rigamonti C Andorno S Maduli E Capelli F Boldorini R Sartori M Gender and liver fibrosis in chronic hepatitis: the role of iron status Aliment Pharmacol Ther 2005 5 1445 1451 10.1111/j.1365-2036.2005.02517.x 15948811

[B138] Roderburg C Urban GW Bettermann K Vucur M Zimmermann H Schmidt S Janssen J Koppe C Knolle P Castoldi M Tacke F Trautwein C Luedde T Micro-RNA profiling reveals a role for miR-29 in human and murine liver fibrosis Hepatology 2011 5 209 218 10.1002/hep.23922 20890893

